# Cave‐dwelling bats of Carajás National Forest: New cytogenetic data of threatened species

**DOI:** 10.1002/ece3.11296

**Published:** 2025-04-21

**Authors:** Jéssica Barata da Silva, Thayse Cristine Melo Benathar, Ramerson Lucas Ferreira Azevedo, Leonardo Carreira Trevelin, Cleusa Yoshiko Nagamachi, Guilherme Oliveira, Julio Cesar Pieczarka

**Affiliations:** ^1^ Laboratório de Citogenética, Centro de Estudos Avançados da Biodiversidade, Instituto de Ciências Biológicas Universidade Federal do Pará Belém Brazil; ^2^ Instituto Tecnológico Vale Belém Brazil

**Keywords:** biodiversity, Chiroptera, cytotaxonomy, karyotype, underground ecosystems

## Abstract

In Brazil, bat species that use caves as shelters are constantly susceptible to anthropic impacts. Of the 181 species of bats in Brazil, 81 are recorded in caves, 13 of which are considered essentially cave dwellers. The Carajás National Forest Conservation Unit, located in the southeastern region of Pará, serves as a refuge for these species, among others. The present work sought to reveal the chromosomal diversity of bats in six caves of the Carajás National Forest. Cytogenetic analyses were carried out for some species as an additional tool for taxonomic identification. We present here the conventional karyotype of eight species, five being described for the first time in Brazilian specimens. We also described for the first time the karyotypes of *Natalus macrourus* and *Peropteryx kappleri*. These findings may be helpful to support conservation guidelines, and for the knowledge of these species' biodiversity, evolutionary history, and to support genome sequencing efforts.

## Graphical abstract

The Carajás National Forest, a Conservation Unit protected by law, is one of the few forest remnants in the southeastern region of Pará, Eastern Amazonia, Brazil, reconciling biodiversity protection with economic activities (i.e. mining). The present study aims to describe the chromosomal diversity of bats in natural caves of the Carajás National Forest. Our results allow the chromosomal characterization of the collected taxa, helping to define their taxonomic status and thus contributing to decision‐making about the management of the region, from the point of view of biodiversity conservation.

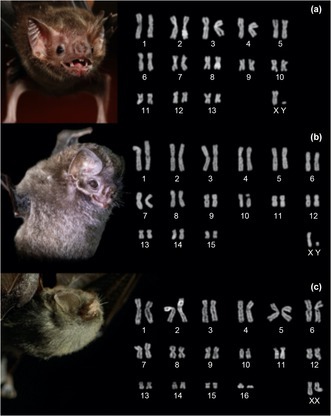

## 1 INTRODUCTION

Underground ecosystems are widely found in the Neotropical region (Gnaspini & Trajano, 2000; Ladle et al., 2012). Brazil is rich in caves, with an estimated 310,000 such environments in the Brazilian territory, although <5% have been officially recorded (Piló & Auler, 2011). In a given diversity of subterranean environments, many organisms inhabit them (Kunz, 1982).

Caves are important as shelters for bats because they provide a favorable environment for the ecological and physiological maintenance of these mammals (De Sousa Barros et al., 2020). Many bat species are dependent on subterranean ecosystems, as they provide protection against predation, act as sites of reproduction and mating, and facilitate social exchange and the adaptive maintenance of body temperature during torpor or hibernation (De Sousa Barros et al., 2020; Furey & Racey, 2015; Kunz, 1982).

Brazil has comparatively strict legislation protecting underground ecosystems (and bat caves), where environmental licensing processes are required for any economic activity with the potential to impact these ecosystems (Brasil). However, the Brazilian government deals with pressures to make this protective legislation more flexible, which could threaten diverse populations of fauna and flora and the ecosystem services they provide.

The southeastern region of Pará state (Brazilian east Amazonian region) has a wide history of anthropogenic environmental interference, leaving only fragments of forest remnants (Souza‐Filho et al., 2016). The Carajás region, where there is the largest iron ore mine in the world, is home to approximately 1000 caves, being of great speleological importance (Piló et al., 2015). The Carajás National Forest, where many of these caves are located, is a conservation unit that presents a wide range of outstanding landforms and is one of the most important regions in the Brazilian Amazon (Piló et al., 2015). Its management plan establishes areas for scientific research, conservation, and mining, among other uses (Martins et al., 2012). In addition to the regional richness in subterranean environments, the Carajás National Forest is a portion of the Amazon biome composed of a mosaic of dominant vegetation types. Where caves are surrounded by the savanna physiognomy of canga vegetation, such caves are the main stable shelter for bats (Martins et al., 2012).

Caves must be evaluated as nodes in a network of underground environments in karstic landscapes. Therefore, the evaluation and relevance classification of these environments in environmental studies need to consider a broader scale (Delgado‐Jaramillo et al., 2018; Jaffe et al., 2016). Studies aimed at understanding the genetic diversity in bioindicator taxa can help to demonstrate the connectivity between subterranean environments and guide management and conservation decisions.

Of the 181 bat species in Brazil, 81 were recorded in caves and two of the four endangered species (Barros & Bernard, 2023) are dependent on these locations (De Oliveira et al., 2018; Garbino et al., 2020). Conservation strategies for these environments depend on knowledge of species distribution, diversity patterns, levels of endemism, and taxonomy (Moreira et al., 2009). However, the scarcity of knowledge, mainly due to the lack of comprehensive inventories and low taxonomic resolution of the available data, are the main obstacles to proposing conservation measures (Gallão & Bichuette, 2015).

Karyotype can be thought of as a map of the nuclear genome. Far from being only a descriptive branch of knowledge, cytogenetics is valuable as a powerful tool that contributes to bat systematics and taxonomy (Rieseberg, 2001; Sotero‐Caio et al., 2017). As most mammals' species show a species‐specific karyotype, descriptions of karyotypes serve an important role in species characterization and also in defining chromosomal rearrangements, which can provide information about genetic barriers and the processes involved in speciation (Baker, 1967; Faria & Navarro, 2010). In this regard, investigative chromosome studies can provide evidence of karyotypic variation of poorly studied species, such as the threatened cave‐dwelling bat species. Therefore, describing the chromosomal diversity in sensitive environments such as caves and documenting the genetic variation of endangered species are useful tools integrated with other approaches in conservation studies.

Cytogenetic studies in South American bats have a long history, since the 1960s (Baker, 1967), but only in this century the chromosome panting research began (Pieczarka et al., 2005). So, most of the literature on South American bat's karyotypes is on classical cytogenetics analysis. Many species still do not have their karyotypes described. This is especially relevant because cytogenetic information on bat species that inhabit difficult‐to‐access environments, such as caves, is scarce (Table 1).

**TABLE 1:** Cytogenetic data available in the literature and obtained in the present study.

Family	Species	2*n*	FNa	Locality	References	
Phyllostomidae	*Diphylla ecaudata*	32	60	Pernambuco, Brazil	Santos et al. (2001) and Sotero‐Caio et al. (2011)	
Phyllostomidae	*Desmodus rotundus*	28	52	Pernambuco, Brazil	Santos et al. (2001) and Sotero‐Caio et al. (2011)	
	*Lonchorhina aurita*	32	60	Nickerie, Suriname	Baker et al. (1981), Farias et al. (2021) and Barros et al. (2009)	
Furipteridae	*Furipterus horrens*	34	62	Saramacca, Suriname	Baker et al. (1981)	
Furipteridae	*Furipterus horrens*	34	60	Carajas, Brazil	Present study	
Vespertilionidae	*Myotis albescens*	44	50	Huánuco, Peru	Bickham et al. (1986)	
Natalidae	*Natalus macrourus*	36	54	CNF, Parauapebas, Pará, Brazil	Present study	
Emballonuridae	*Peropteryx kappleri*	26	48	CNF, Parauapebas, Pará, Brazil	Present study	
Mormoopidae	*Pteronotus personatus*	38	60	Veracruz, Mexico	Farias et al. (2021) and Sites Jr et al. (1981)	

Abbreviation: CNF, Carajás National Forest.

In this paper we report the results of a cytogenetics study of bats obtained from six caves, aiming to describe their chromosomal diversity in natural caves in the Carajás National Forest. We provided karyotypes of eight important species for the cave ecosystem, two of than classified in the IUCN red list as Least Concern (*Furipterus horrens*), and Not Assessed (*Natalus macrourus*) (IUCN). We described for the first time the karyotype of *N. macrourus*, an important species that is threatened. We compared our data with previously published data from the same or closely related species, to verify possible karyotypic variations.

## 2 MATERIALS AND METHODS

### 2.1 Study area

**FIGURE 1:**
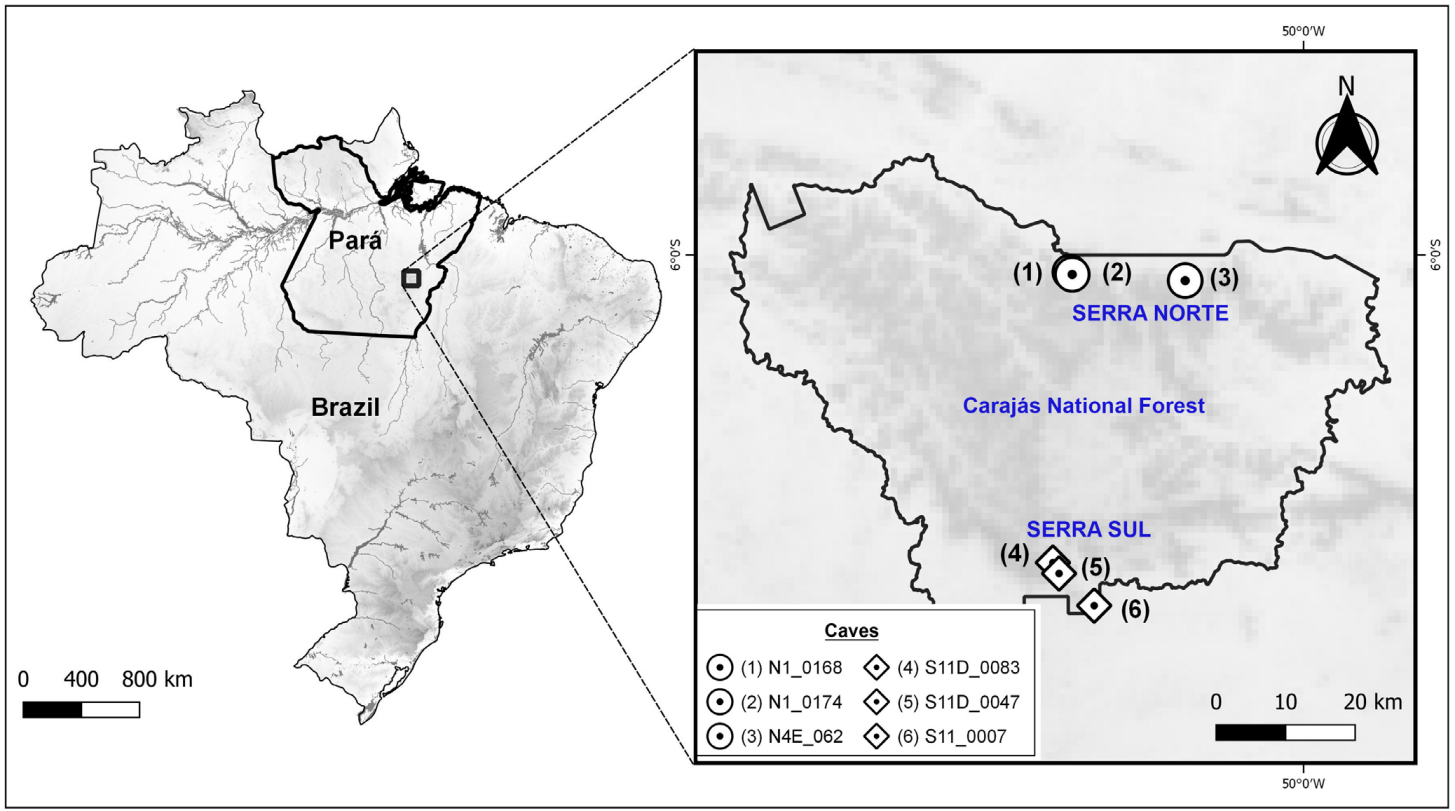
Location of the study region and zoom of the study area showing the spatial distribution of the sampled caves, whose symbols indicate localities in the “Serra Norte” and “Serra Sul” places. The numbers refer to localities in Table 2.

This study was carried out in Carajás National Forest (CNF), which is in the southeastern region of the State of Pará in domains of the Itacaiúnas River basin, affluent of the Tocantins River, whose confluence occurs in the city of Marabá (Figure 1). CNF occupies an area of 411,949 ha, in lands in the municipalities of Parauapebas, Canaã dos Carajás and Água Azul do Norte (Campos & Castilho, 2012). The map was made using QGIS v.3.10.7. The shapefiles containing geographic data (elevation, hydrography, and country limits) were obtained from DIVA‐GIS (Hijmans et al., 2004). We used the limits provided by Braga et al. (2008) and we created the shapefiles on QGIS v.3.10.7.

### 2.2 Collected material, identification, and ethic statements

We collected bats in two expeditions of three consecutive days in November 2021 and March 2022 that visited three caves in Serra Norte and three in Serra Sul. Captures were conducted using harp traps placed at the caves' entrance and Ecotone Inc. 12 × 3‐m mist nets outside, in the understory of nearby forests. Details of cave names are in Table 2. The traps were open in the late afternoon and were closed after 4 h of sampling.

**TABLE 2:** Collection sites in Carajás National Forest (Pará, Brazil), with the respective sample sizes (Arita, 1993).

Species	Individuals/sex	Classification	Locality	Cave	Geographic Coordinate (datum WGS‐84)
*Diphylla ecaudata*	1♂	EC	Serra Sul	S11_0007 (6)	−50.242, −6.456
*Desmodus rotundus*	1♂	OC	Serra Sul	S11_0007 (6)	−50.242, −6.456
*Lonchorhina aurita*	1♀ 1♂	EC	Serra Norte	N1_0168 (1)	−50.302, −6.022
*Furipterus horrens*	1♀	EC	Serra Sul	S11_0007 (6)	−50.242, −6.456
*Myotis albescens*	1♂	OC	Serra Norte	N1_0168 (1)	−50.302, −6.022
*Natalus macrourus*	1♂ 1♀	EC	Serra Sul	S11D_0083 (4)	−50.324, −6.397
*Peropteryx kappleri*	1♀ 1♂	OC	Serra Norte	N4E_062 (3) S11D_0047 (5)	−50.153, −6.033
*Pteronotus personatus*	1♀	EC	Serra Norte	N1_0174 (2)	−50.299, −6.025

Abbreviations: EC, essentially cavernicolous; OC, occasionally cavernicolous.

The captures were authorized by the Brazilian Environment Department under license (IBAMA). JCP has a permanent field permit (number 13248) and a special field permit for collecting samples of cave biodiversity (number 76429) from “Instituto Chico Mendes de Conservação da Biodiversidade”. The Cytogenetics Laboratory from UFPA has a special permit number 19/2003 from the Ministry of Environment for sample transport and 52/2003 for using the samples for research. We followed the guidelines for the use of wild mammals in research (Sikes, 2016), collection, and documentation of specimens (Pitt, 2008). The sample sacrifice was performed by animal welfare guidelines established by Brazilian resolution CFMV n.1000/2012 and in accordance with animal welfare guidelines established by the Animal Ethics Committee (Comitê de Ética Animal) from Universidade Federal do Pará (Permit 68‐2015).

### 2.3 Samples

Eleven specimens were collected (Table 2) and the vouchers were deposited at the mammal collection of Museu Paraense Emílio Goeldi (MPEG). Each captured bat was identified according to their morphological features and external and craniodental measurements using keys and other available literature describing these species (Gardner, 2008; Simmons, 2005). Bats were classified considering classification as essentially cavernicolous (EC) and occasionally cavernicolous (OC), concerning shelter use strategies of caves (Arita, 1993).

### 2.4 Chromosomal preparations and karyotype analysis

Chromosomal preparations were obtained through direct bone marrow extraction, according to the procedure described (Baker et al., 2003) and primary culture of fibroblasts (Moratelli et al., 2002) with minor adjustments. A 5% Giemsa in phosphate buffer solution was used to stain the metaphases. For karyotype description and diploid number, 10 metaphases were analyzed per species. The best metaphases were selected for karyotyping. An Olympus BX41 microscope and a CCD 1300QDS digital camera were used to obtain digital images from conventional staining, which were analyzed using the GenA‐SIs software v. 7.2.7.34276.

## 3 RESULTS

**FIGURE 2:**
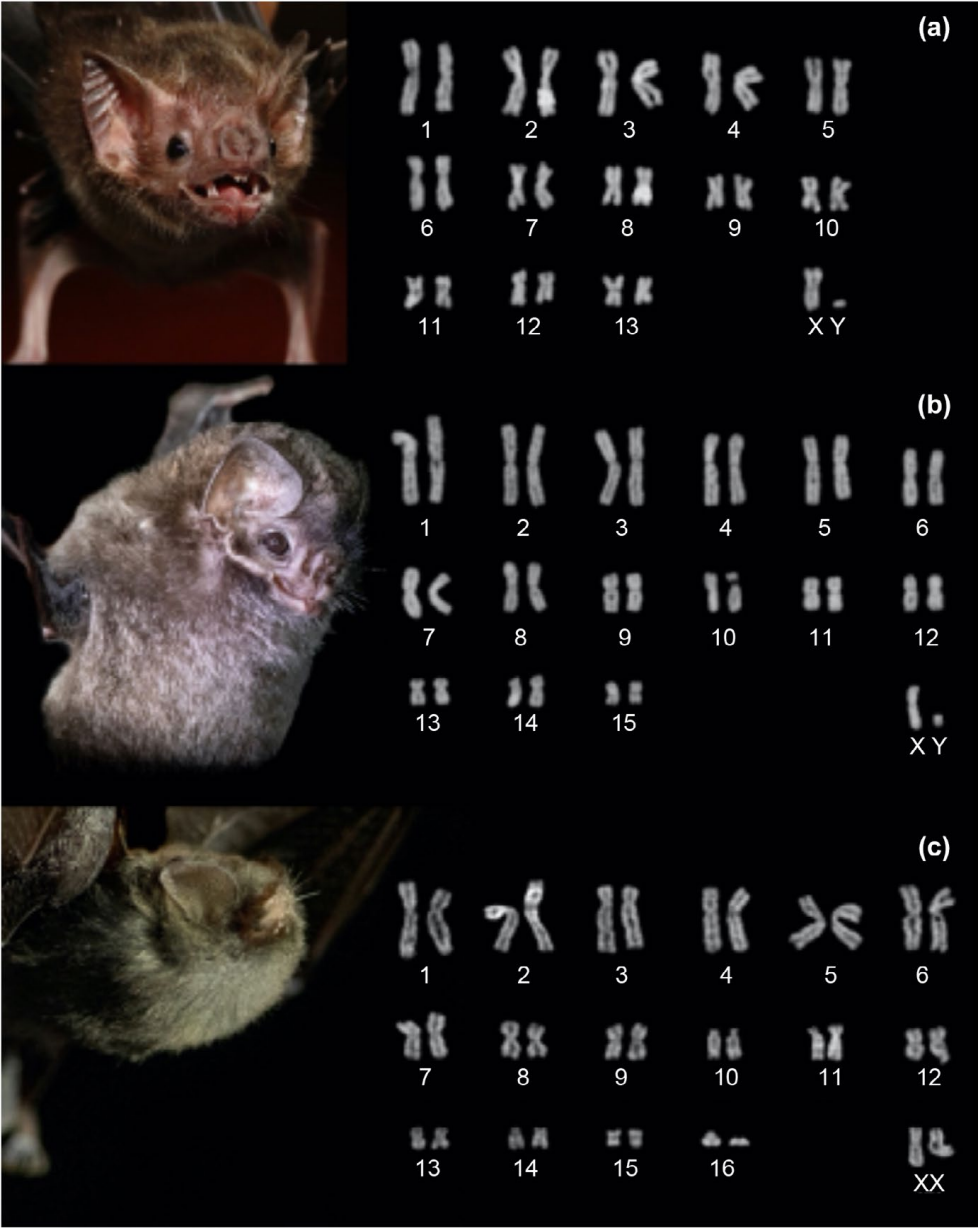
Conventional Giemsa staining of chromosomes of selected species from Carajás National Forest. (a) *Desmodus rotundus* (2*n* = 28, FNa = 52); (b) *Diphylla ecaudata* (2*n* = 32, FNa = 60); (c) *Furipterus horrens* (2*n* = 34, FNa = 60). The Giemsa‐stained chromosomes had their color inverted.

**FIGURE 3:**
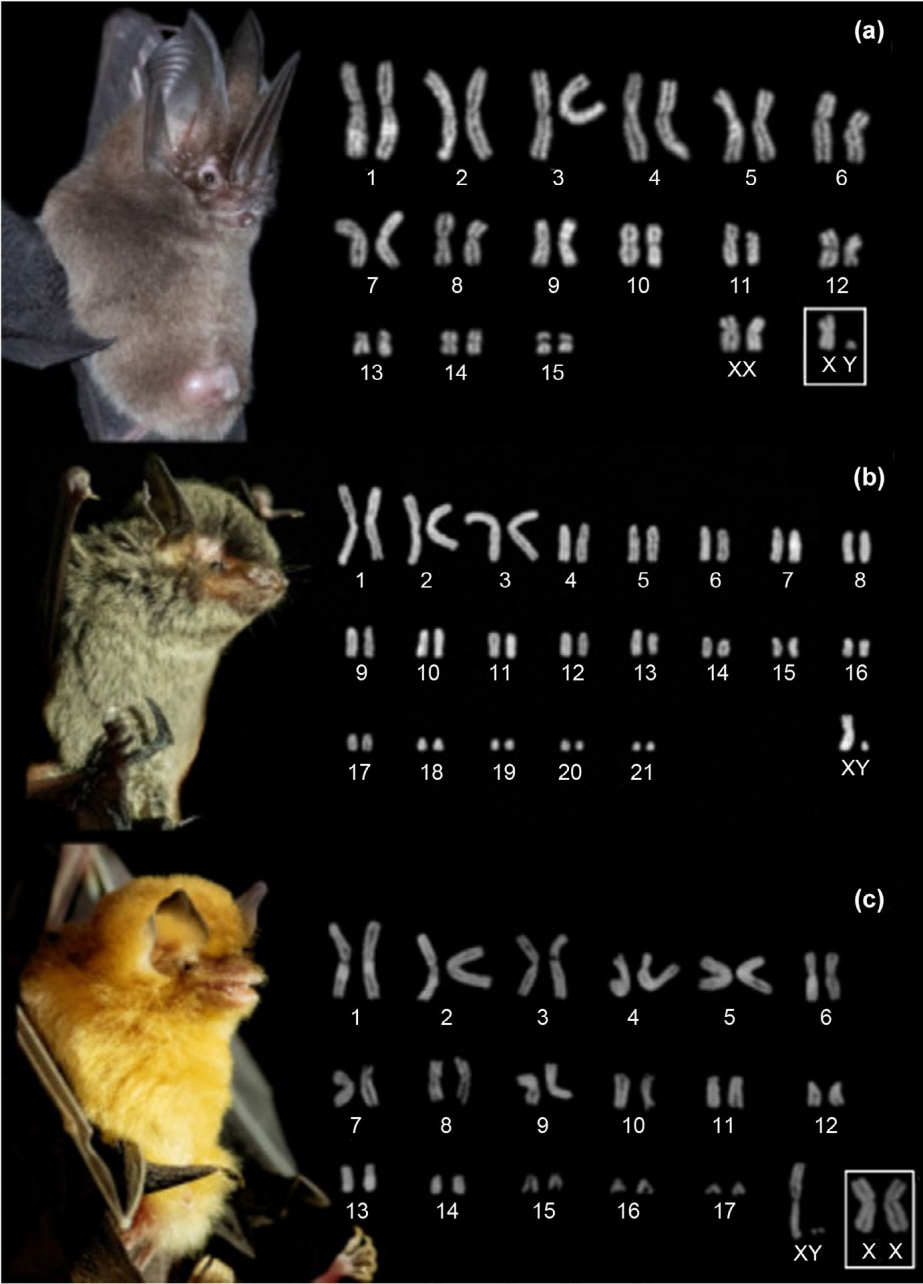
Conventional Giemsa staining of chromosomes of selected species from Carajás National Forest. (a) *Lonchorhina aurita* (2*n* = 32, FNa = 60); (b) *Myotis albescens* (2*n* = 44, FNa = 50); (c) *Natalus macrourus* (2*n* = 36, FNa = 56). Chromosome pair into a square: sex chromosomes from another specimen. The Giemsa‐stained chromosomes had their color inverted.

We described diploid chromosome number (2*n*), and autosomal fundamental number (FNa) of a sample of 11 specimens of eight species. The male specimen of *Desmodus rotundus* has 2*n* = 28, FNa52, and all autosomes had meta/submetacentric morphology; the X is a mid‐sized submetacentric and Y is a punctiform chromosome (Figure 2a). The karyotype of *Diphylla ecaudata* shown 2*n* = 32, FNa = 60, all autosomes had meta/submetacentric morphology, and chromosome X is submetacentric and Y is a small submetacentric (Figure 2b). The karyotype of a female *F. horrens* shows 2*n* = 34, FNa = 60, composed of 14 pairs of meta/submetacentric chromosomes (1–11, 13, 16–17) and two pairs of acrocentric chromosomes (10, 16); since no banding was done, we supposed that the X is a mid‐sized submetacentric (Figure 2c). The male and female specimens of *Lonchorhina aurita* have 2*n* = 32, FNa = 60, all autosomes had meta/submetacentric chromosomes; chromosome X is submetacentric and Y is small acrocentric (Figure 3a).

**FIGURE 4:**
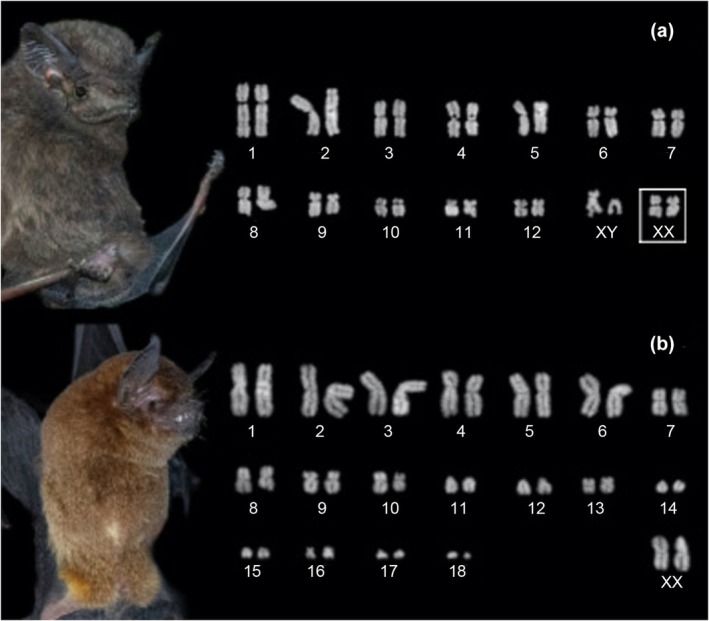
Conventional Giemsa staining of chromosomes of selected species from Carajás Nacional Forest. (a) *Peropteryx kappleri* (2*n* = 26, FNa = 48); (b) *Pteronotus personatus* (2*n* = 38, FNa = 60). Chromosome pair into a square: sex chromosomes from another specimen. The Giemsa‐stained chromosomes had their color inverted.

The karyotype of *Myotis albescens* shown 2*n* = 44, FNa = 50, composed of four pairs of meta/submetacentric (1–3, 16) and 17 acrocentric chromosomes (4–15, 17–21); the chromosome X is submetacentric and Y is small acrocentric (Figure 3b). The analyzed *N. macrourus* specimens presented 2*n* = 36, FNa = 56, with 11 pairs meta/submetacentric chromosomes (1–9, 13) and 6 pairs acrocentric chromosomes (10–12, 14–17); the chromosome X is submetacentric and Y is small acrocentric (Figure 3c). A male specimen of *Peropteryx kappleri* has 2*n* = 26, FNa = 48; all autosomes had meta/submetacentric morphology; the X is a submetacentric chromosome and Y is acrocentric about half the size of the X‐chromosome (Figure 4a). Finally, the karyotype of *Pteronotus personatus* presented 2*n* = 38, FNa = 60, composed of 12 meta/submetacentric chromosome pairs (1–10, 13, 16) and 6 acrocentric pairs (11–12, 14–15, 17–18); the chromosome X is mid‐sized metacentric (Figure 4b).

## 4 DISCUSSION

The Carajás National Forest is an excellent target for studying bats because it consists of an area of high biological and landscape diversity, harboring over 1000 caves, one of the largest concentrations of caves in Brazil (Martins et al., 2012; Piló et al., 2015). Of the 23 bat species registered in the CNF caves (Tavares et al., 2012), our study revealed a representativeness of **~**35% of this diversity. Furthermore, the reported data on bat occurrence in 269 Brazilian caves classified the species as essentially cavernicolous, opportunistic cavernicolous, and non‐cavernicolous (Guimarães & Ferreira, 2014). In the present study, over half of the karyotyped species investigated are essentially cavernicolous (Table 1).

It is essential to improve the representativeness of cave bats´ cytogenetic data since <5% of the caves in Brazil have been comprehensively explored (Jansen et al., 2012). Furthermore, most studies have focused on areas other than the Brazilian Amazon, leaving the region with <1% of its known cave bat population investigated (Cajaiba et al., 2021; De Oliveira et al., 2018; Guimarães & Ferreira, 2014; Sites Jr et al., 1981). This representatively is further worrying, as Amazonian caves show more variable bat assemblage compositions than caves in other regions of Brazil (De Oliveira et al., 2018).

The karyotypes recovered from our samples were similar to those already published (see Table 1), however, there was no previous knowledge of the karyotypes of Brazilian populations of *F. horrens*, *M. albescens*, *N. macrourus*, *P. kappleri* and *P. personatus*. Our sample of *F. horrens* has a divergent karyotype concerning chromosome structure compared to the karyotyped specimen from Suriname (Baker et al., 1981). In the present study, *F. horrens* was found to have a karyotype of 2*n* = 34/FN = 60, while in the previous description the FNa = 62 (Baker et al., 1981). The difference results from the fact that pair 10 is acrocentric in our karyotype while is subtelocentric in the previous study (Baker et al., 1981). However, in both studies, the sexual chromosome pair was not different since in both situations only females were analyzed, so it is possible that the FNa described is different from that suggested. The same happens here since we are not sure that the pair designated as X is the sex chromosome.

*Natalus* is an infrequently encountered species and is threatened by the practice of extermination of cave bat colonies (e.g., in campaigns against rabies) (Tejedor, 2011; Tejedor et al., 2005). The genus *Natalus* has a complex taxonomic history and studies based on morphological and molecular characters indicate cryptic diversity in some species (López‐Wilchis et al., 2012). Currently, eight valid species with Neotropical distribution are recognized (Tejedor, 2011). In Brazil, there was some uncertainty in the classification, as *N. stramineus* has been historically applied to populations of the genus *Natalus* from virtually the entire Neotropics. Later the populations in the south of the Amazon River were classified as *N. espiritosantensis*, and the samples of the North, in the shields of the Guyanas as *N. tumidirostris* (Garbino & Tejedor, 2013). As *N. macrourus* was considered a senior synonym of *N. espiritosantensis* (Garbino & Tejedor, 2013), this became the valid name of the species occurring south of the Amazon River.

Baker (1970) described the karyotype of what is supposed to be *Natalus stramineus*, from populations in Mexico with 2*n* = 36, FN = 56. Assessing the geographic location of the reported studies, the karyotype of the populations of Mexico is *N. mexicanus* or *N. lanatus* (however, some authors disagree that there are two species of *Natalus* in Mexico, see (López‐Wilchis et al., 2012)), since after review Baker and Jordan (1970) and Tejedor (2011) characterized *N. tumidirostris* from Trinidad and Tobago with the same chromosome number and morphology. However, *N. stramineus* is reported only in the northern Lesser Antilles. So, the described karyotype from Trinidad and Tobago may be *N. stramineus*. By their turn, Volleth et al. (2023) described the karyotypes of *N. mexicanus* from Cueva de las Vegas, Tenampulco, Mexico having 2*n* = 36 and FNa = 56, and *N. tumidirostris* from Candelaria, Venezuela, as having also 2*n* = 36 and FNa = 56. Those authors state that 2*n* = 36 and FNa = 56 is the most common form, although some authors describe FNa = 54. To summarize, due to this extensive history of changes in the taxonomy of *Natalus*, it was assumed that we already knew the karyotype of the species that is distributed in the Brazilian Amazon (Dos Reis et al., 2007), but this was not true until the present work. The karyotype here described is similar to the others previously described, with 2*n* = 36, and FN = 56. This means that the speciation in this genus is related to geographic isolation. The populations must be large, with a reasonable ability to travel over wide regions, avoiding inbreeding (Dobigny et al., 2015).

Although *Natalus* species have the same chromosomal number and morphology, knowledge of the cytogenetics of the group can provide useful data in further studies to differentiate the species from the analysis of repetitive portions of the genome using the FISH (Fluorescence in situ hybridization) technique and assist in the complex evolutionary history of this taxon.

In the present study, we described for the first time the karyotype of *P. kappleri*. This species is not an obligate cave dweller and can also roost in tree hollows and rock crevices (Voss et al., 2016). Previously, cytogenetic data was only available for *P. macrotis*, with a karyotype composed of 2*n* = 26 and FN = 48. The same diploid and fundamental numbers were found in our study, without evidence of chromosomal differences when compared to the congener *P. macrotis* from other Amazonian locations (Baker et al., 1981). The only difference would be that the X chromosome of *P. macrotis* was described as near acrocentric (Baker et al., 1981) while in our study *P. kappleri* has a submetacentric X. Regarding the other species, we demonstrate that the specimens sampled in this study show karyotypes characteristic expected for what is already known in the literature for these species, evidencing taxonomical stability.

## 5 CONCLUSIONS

Cytogenetic data can provide interesting information to help characterize the karyotypes of these species. The karyotypic description has helped in taxonomic studies, including diagnostic characters for delimiting taxa. The study of genetic diversity together with the systematic monitoring of bat populations in the Carajás National Forest region are essential tools in the conservation of these important indicators of biodiversity and the maintenance of cave ecosystems.

## AUTHOR CONTRIBUTIONS

**Jéssica Barata da Silva:** Conceptualization (lead); data curation (lead); formal analysis (equal); writing – original draft (equal). **Thayse Cristine Melo Benathar:** Formal analysis (supporting); methodology (equal); writing – review and editing (equal). **Ramerson Lucas Ferreira Azevedo:** Formal analysis (supporting); methodology (equal); writing – review and editing (supporting). **Leonardo Carreira Trevelin:** Data curation (equal); funding acquisition (supporting); investigation (equal); resources (supporting); writing – review and editing (equal). **Cleusa Yoshiko Nagamachi:** Data curation (equal); funding acquisition (equal); investigation (equal); resources (equal); writing – review and editing (equal). **Guilherme Oliveira:** Data curation (equal); funding acquisition (supporting); investigation (equal); resources (supporting); writing – review and editing (equal). **Julio Cesar Pieczarka:** Project administration (lead); resources (lead); supervision (lead); writing – review and editing (equal).

## FUNDING INFORMATION

This research was funded by the Instituto Brasileiro de Desenvolvimento e Sustentabilidade (IABS), grant number 010/2020; by the Conselho Nacional de Desenvolvimento Científico e Tecnológico, grants number 307170/2021‐7 and 307154/2021‐1; and by the Banco Nacional de Desenvolvimento Econômico e Social, grant number 2.318.697.0001.

## CONFLICT OF INTEREST STATEMENT

The authors declare no conflict of interest.

## INSTITUTIONAL REVIEW BOARD STATEMENT

The animal study protocol was approved by the Ethics Committee (Comitê de Ética Animal) from Universidade Federal do Pará (Permit 68‐2015), which also approved all experimental protocols of this research. The captures were authorized by the Brazilian Environment Department under license (IBAMA 02047.000384/2007‐34). JCP has a permanent field permit (number 13248) and a special field permit for collecting samples of cave biodiversity (number 76429) from “Instituto Chico Mendes de Conservação da Biodiversidade”.

## Data Availability

All data used in this research are available in the article. The authors are available for any further explanation.

## References

[ece311296-bib-0001] Arita, H. T. (1993). Conservation biology of the cave bats of Mexico. Journal of Mammalogy, 74(3), 693–702.

[ece311296-bib-0002] Baker, R. (1967). Karyotypes of bats of the family Phyllostomidae and their taxonomic implications. The Southwestern Naturalist, 12(4), 407–428.

[ece311296-bib-0003] Baker, R. J. (1970). Karyotypic trends in bats. Biology of Bats, 1, 65–95.

[ece311296-bib-0004] Baker, R. J. , Genoways, H. H. , & Seyfarth, P. A. (1981). Results of the Alcoa Foundation‐Suriname expeditions. VI. Additional chromosomal data for bats (Mammalia: Chiroptera) from Suriname. Mammalogy Papers: University of Nebraska State Museum, 230, 333–344.

[ece311296-bib-0005] Baker, R. J. , Hamilton, M. J. , & Parish, D. A. (2003). Preparations of mammalian karyotypes under field conditions. Occasional Papers, Museum of Texas Tech University, 228, i+1–8.

[ece311296-bib-0006] Baker, R. J. , & Jordan, R. G. (1970). Chromosomal studies of some Neotropical bats of the families Emballonuridae, Noctilionidae, Natalidae and Vespertilionidae. Caryologia, 23(4), 595–604.

[ece311296-bib-0007] Barros, H. M. D. R. , Sotero‐Caio, C. G. , Santos, N. , & de Souza, M. J. (2009). Comparative cytogenetic analysis between *Lonchorhina aurita* and *Trachops cirrhosus* (Chiroptera, Phyllostomidae). Genetics and Molecular Biology, 32(4), 748–752. 21637449 10.1590/S1415-47572009005000095 PMC3036889

[ece311296-bib-0008] Barros, J. S. , & Bernard, E. (2023). Species richness, occurrence and rarity of bats in Brazilian caves. Austral Ecology, 48, 2144–2170. 10.1111/aec.13453

[ece311296-bib-0009] Bickham, J. W. , McBee, K. , & Schlitter, D. A. (1986). Chromosomal variation among seven species of *Myotis* (Chiroptera: Vespertilionidae). Journal of Mammalogy, 67(4), 746–750.

[ece311296-bib-0010] Braga, B. P. F. , Flecha, R. , Pena, D. S. , & Kelman, J. (2008). Pacto federativo e gestão de águas. Dossiê Água. Estudos Avançados, 22(63), 17–42. 10.1590/S0103-40142008000200003

[ece311296-bib-0011] Brasil . Decreto n° 10.935, 12 de janeiro de 2022. Diário Oficial da República Federativa do Brasil. Ano CLX N° 8‐A, (01): 01. https://pesquisa.in.gov.br/imprensa/jsp/visualiza/index.jsp?jornal=600&pagina=1&data=12/01/2022&totalArquivos=6

[ece311296-bib-0012] Cajaiba, R. L. , Périco, E. , da Silva, W. B. , Vieira, T. B. , dos Santos, F. M. B. , & Santos, M. (2021). Are neotropical cave‐bats good landscape integrity indicators? Some clues when exploring the cross‐scale interactions between underground and above‐ground ecosystems. Ecological Indicators, 122, 107258.

[ece311296-bib-0013] Campos, J. F. , & Castilho, A. (2012). Uma Visão Geográfica da Região da Flona de Carajás. In F. D. Martins , A. F. Castilho , J. F. Campos , F. M. Hatano , & S. G. Rolim (Eds.), Floresta Nacional de Carajás: Estudos sobre vertebrados terrestres (Vol. 1, 1st ed., pp. 28–63). Nitro Imagens.

[ece311296-bib-0015] De Oliveira, H. F. M. , Oprea, M. , & Dias, R. I. (2018). Distributional patterns and ecological determinants of bat occurrence inside caves: A broad scale meta‐analysis. Diversity, 10(3), 49.

[ece311296-bib-0016] De Sousa Barros, J. , Bernard, E. , & Ferreira, R. L. (2020). Ecological preferences of Neotropical cave bats in roost site selection and their implications for conservation. Basic and Applied Ecology, 45, 31–41.

[ece311296-bib-0017] Delgado‐Jaramillo, M. , Barbier, E. , & Bernard, E. (2018). New records, potential distribution, and conservation of the near threatened cave bat *Natalus macrourus* in Brazil. Oryx, 52(3), 579–586.

[ece311296-bib-0018] Dobigny, G. , Britton‐Davidian, J. , Terence, J. , & Robinson, T. J. (2015). Chromosomal polymorphism in mammals: An evolutionary perspective. Biological Reviews, 92, 1–29. 10.1111/brv.12213 26234165

[ece311296-bib-0019] Dos Reis, N. R. , Peracchi, A. L. , Pedro, W. A. , & de Lima, I. P. (2007). Morcegos do Brasil. Universidade Estadual de Londrina.

[ece311296-bib-0020] Faria, R. , & Navarro, A. (2010). Chromosomal speciation revisited: Rearranging theory with pieces of evidence. Trends in Ecology & Evolution, 25(11), 660–669. 20817305 10.1016/j.tree.2010.07.008

[ece311296-bib-0021] Farias, J. C. , Santos, N. , Bezerra, D. P. , & Sotero‐Caio, C. G. (2021). Chromosome painting in *Lonchorhina aurita* sheds light onto the controversial phylogenetic position of sword‐nosed bats (Chiroptera, Phyllostomidae). Cytogenetic and Genome Research, 161(12), 569–577. 35093945 10.1159/000520969

[ece311296-bib-0022] Furey, N. , & Racey, P. A. (2015). Conservation ecology of cave bats. In C. C. Voigt & T. Kingston (Eds.), Bats in the Anthropocene: Conservation of bats in a changing world (Vol. 1, 1st ed., pp. 463–500). Springer International Publishing.

[ece311296-bib-0023] Gallão, J. E. , & Bichuette, M. E. (2015). Taxonomic distinctness and conservation of a new high biodiversity subterranean area in Brazil. Anais da Academia Brasileira de Ciências, 87, 209–217. 10.1590/0001-3765201520140312 25673471

[ece311296-bib-0024] Garbino, G. S. , & Tejedor, A. (2013). *Natalus macrourus* (Gervais, 1856) (Chiroptera: Natalidae) is a senior synonym of *Natalus espiritosantensis* (Ruschi, 1951). Mammalia, 77(2), 237–240.

[ece311296-bib-0025] Garbino, G. S. T. , Gregorin, R. , Lima, I. P. , Loureiro, L. , Moras, L. M. , Moratelli, R. , Nogueira, M. R. , Pavan, A. C. , Tavares, V. C. , Nascimento, M. D. , & Peracchi, A. L. (2020). Updated checklist of Brazilian bats. Comitê da Lista de Morcegos do Brasil—CLMB. Sociedade Brasileira Para o Estudo de Quirópteros (Sbeq). https://www.sbeq.net/lista‐de‐especies

[ece311296-bib-0026] Gardner, A. L. (2008). Mammals of South America, volume 1: Marsupials, xenarthrans, shrews, and bats (1st ed.). University of Chicago Press. 10.7208/chicago/9780226282428.001.0001

[ece311296-bib-0027] Gnaspini, P. , & Trajano, E. (2000). Guano communities in tropical caves. In H. Wilkens , D. C. Culver , & W. F. Humphreys (Eds.), Ecosystems of the world. Subterranean ecosystems (1st ed., pp. 251–268). Elsevier.

[ece311296-bib-0028] Guimarães, M. M. , & Ferreira, R. L. (2014). Cave bats in Brazil: New records and conservation challenges. Revista Brasileira de Espeleologia, 2, 1–33.

[ece311296-bib-0029] Hijmans, R. J. , Guarino, L. , Bussink, C. , Mathur, P. , Cruz, M. , Barrentes, I. , et al. (2004). DIVA‐GIS. Vns5.0. A geographic information system or the analysis of species distribution data. http://www.diva‐gis.org

[ece311296-bib-0030] IUCN . The red list of threatened species. Version 2023‐1. https://www.iucnredlist.org/

[ece311296-bib-0031] Jaffe, R. , Prous, X. , Zampaulo, R. , Giannini, T. C. , Imperatriz‐Fonseca, V. L. , Maurity, C. , Oliveira, G. , Brandi, I. V. , & Siqueira, J. O. (2016). Reconciling mining with the conservation of cave biodiversity: A quantitative baseline to help establish conservation priorities. PLoS One, 11(12), e0168348. 27997576 10.1371/journal.pone.0168348 PMC5173368

[ece311296-bib-0032] Jansen, D. C. , Cavalcanti, L. F. , & Lamblém, H. S. (2012). Mapa de potencialidade de ocorrência de cavernas no Brasil, na escala 1:2.500.000. Revista Brasileira de Espeleologia, 2, 42–57.

[ece311296-bib-0033] Kunz, T. H. (1982). Roosting ecology of bats. In T. H. Kunz (Ed.), Ecology of bats (1st ed., pp. 1–55). Plenum Press.

[ece311296-bib-0034] Ladle, R. J. , Firmino, J. V. L. , Malhado, A. C. M. , & Rodríguez‐Durán, A. (2012). Unexplored diversity and conservation potential of Neotropical hot caves. Conservation Biology, 26(6), 978–982. 23003344 10.1111/j.1523-1739.2012.01936.x

[ece311296-bib-0035] López‐Wilchis, R. , Guevara‐Chumacero, L. M. , Pérez, N. Á. , Juste, J. , Ibáñez, C. , & Sosa, I. B. D. (2012). Taxonomic status assessment of the Mexican populations of funnel‐eared bats, genus *Natalus* (Chiroptera: Natalidae). Acta Chiropterologica, 14(2), 305–316.

[ece311296-bib-0036] Martins, F. D. , Castilho, A. F. , Campos, J. , Hatano, F. , & Rolim, S. G. (2012). Fauna da Floresta Nacional de Carajás (1st ed., p. 116). Nitro Editorial.

[ece311296-bib-0037] Moratelli, R. , Andrade, C. M. , & Armada, J. L. A. (2002). A technique to obtain fibroblast cells from skin biopsies of living bats (Chiroptera) for cytogenetic studies. Genetics and Molecular Research, 2, 128–130. 14963838

[ece311296-bib-0038] Moreira, J. C. , Manduca, E. G. , Gonçalves, P. R. , Morais, M. M., Jr. , Pereira, R. F. , Lessa, G. , & Dergam, J. A. (2009). Small mammals from Serra do Brigadeiro State Park, Minas Gerais, southeastern Brazil: Species composition and elevational distribution. Arquivos Do Museu Nacional, 67(1–2), 103–118.

[ece311296-bib-0039] Pieczarka, J. C. , Nagamachi, C. Y. , O'Brien, P. C. M. , Yang, F. , Rens, W. , Barros, R. M. S. , Noronha, R. C. R. , Rissino, J. , De Oliveira, E. H. C. , & Ferguson‐Smith, M. A. (2005). Reciprocal chromosome painting between two south American bats: *Carollia brevicauda* and *Phyllostomus hastatus* (Phyllostomidae, Chiroptera). Chromosome Research, 13, 339–347. 15973499 10.1007/s10577-005-2886-0

[ece311296-bib-0040] Piló, L. B. , & Auler, A. (2011). Introdução à Espeleologia. In A. S. Auler , R. Lopes , J. C. R. Reino , & L. B. Piló (Eds.), III Curso de Espeleologia e Licenciamento Ambiental (3rd ed., pp. 7–23). ICMBio/CECAV.

[ece311296-bib-0041] Piló, L. B. , Auler, A. S. , & Martins, F. (2015). Carajás National Forest: Iron ore plateaus and caves in southeastern Amazon. In B. C. Vieira , A. A. R. Salgado , & L. J. C. Santos (Eds.), Landscapes and landforms of Brazil (1st ed., pp. 273–283). Springer.

[ece311296-bib-0042] Pitt, K. E. (2008). Best practices for repositories. Cell Preservation and Technology, 6(1), 1–58.

[ece311296-bib-0043] Rieseberg, L. H. (2001). Chromosomal rearrangements and speciation. Trends in Ecology & Evolution, 16(7), 351–358. 11403867 10.1016/s0169-5347(01)02187-5

[ece311296-bib-0044] Santos, N. , Fagundes, V. , Yonenaga‐Yassuda, Y. , & De Souza Jose, M. (2001). Comparative karyology of Brazilian vampire bats *Desmodus rotundus* and *Diphylla ecaudata* (Phyllostomidae, Chiroptera): Banding patterns, base‐specific fluorochromes and FISH of ribosomal genes. Hereditas, 134(3), 189–194. 11833280 10.1111/j.1601-5223.2001.00189.x

[ece311296-bib-0045] Sikes, R. S. (2016). The animal care and use Committee of the American Society of Mammalogists. 2016 guidelines of the American Society of Mammalogists for the use of wild mammals in research and education. Journal of Mammalogy, 97, 663–688. 10.1093/jmammal/gyw078 29692469 PMC5909806

[ece311296-bib-0046] Simmons, N. B. (2005). Order Chiroptera. In D. E. Wilson & D. M. Reeder (Eds.), Mammal species of the world: A taxonomic and geographic reference (Vol. 1, 3rd ed., pp. 312–529). The Johns Hopkins University Press.

[ece311296-bib-0047] Sites, J. W., Jr. , Bickham, J. W. , & Haiduk, M. W. (1981). Conservative chromosomal change in the bat family Mormoopidae. Canadian Journal of Genetics and Cytology, 23(3), 459–467.

[ece311296-bib-0048] Sotero‐Caio, C. G. , Baker, R. J. , & Volleth, M. (2017). Chromosomal evolution in Chiroptera. Genes, 8(10), 272. 29027987 10.3390/genes8100272 PMC5664122

[ece311296-bib-0049] Sotero‐Caio, C. G. , Pieczarka, J. C. , Nagamachi, C. Y. , Gomes, A. J. B. , Lira, T. C. , O'Brien, P. C. M. , Ferguson‐Smith, M. A. , Souza, M. J. , & Santos, N. (2011). Chromosomal homologies among vampire bats revealed by chromosome painting (Phyllostomidae, Chiroptera). Cytogenetic and Genome Research, 132, 156–164. 21178354 10.1159/000321574

[ece311296-bib-0050] Souza‐Filho, P. W. M. , Souza, E. B. , Silva Júnior, R. O. , Nascimento, W. R., Jr. , Mendonça, B. R. V. , Guimarães, J. T. F. , Dall'Agnol, R. , & Siqueira, J. O. (2016). Four decades of landcover, land‐use and hydroclimatology changes in the Itacaiúnas River watershed, southeastern Amazon. Journal of Environmental Management, 167, 175–184. 26686070 10.1016/j.jenvman.2015.11.039

[ece311296-bib-0051] Tavares, V. C. , Palmuti, C. F. S. , Gregorin, R. , & Dornas, T. T. (2012). Morcegos. In F. D. Martins , A. F. Castilho , J. Campos , F. M. Hatano , & S. G. Rolin (Eds.), Fauna da Floresta Nacional de Carajás: Estudos sobre vertebrados terrestres (1st ed., pp. 162–179). Nitro Editorial.

[ece311296-bib-0052] Tejedor, A. (2011). Systematics of funnel‐eared bats (Chiroptera: Natalidae). Bulletin of the American Museum of Natural History, 353, 1–140.

[ece311296-bib-0053] Tejedor, A. , Tavares, V. C. , & Silva‐Taboada, G. (2005). A revision of extant greater Antillean bats of the genus *Natalus* (Chiroptera: Natalidae). American Museum Novitates, 3493, 1–22.

[ece311296-bib-0054] Volleth, M. , Heller, K. G. , Tidemann, C. , Yong, H. S. , Göpfert, M. , & Müller, S. (2023). Karyotype evolution in Vespertilionoidea: Centromere repositioning and inversions in Molossidae (Chiroptera, Mammalia). Acta Chiropterologica, 25(1), 1–33. https://doi.org/10.31.61/15081109ACC2023.25.1.001

[ece311296-bib-0055] Voss, R. S. , Fleck, D. W. , Strauss, R. E. , Velazco, P. M. , & Simmons, N. B. (2016). Roosting ecology of Amazonian bats: Evidence for guild structure in hyperdiverse mammalian communities. American Museum Novitates, 3870, 1–43.

